# 2822. Impact of Restrictive Urinalysis Reflex to Culture Criteria at a Large Community Hospital

**DOI:** 10.1093/ofid/ofad500.2433

**Published:** 2023-11-27

**Authors:** Christian F Caveness, Alison Orvin, Jeannette Bouchard

**Affiliations:** WakeMed Health and Hospitals, Cary, North Carolina; WakeMed Health and Hospitals, Cary, North Carolina; Prisma Health Richland - University of South Carolina, Columbia, South Carolina

## Abstract

**Background:**

Treatment of asymptomatic bacteriuria (ASB) is a common, but often unnecessary, practice. Some data suggest increased rates of positive urine cultures (UC) lead to ASB treatment. The objective of this study was to determine the impact of restrictive urinalysis reflex to culture (UARC) criteria on rate of UCs ordered and ASB treatment. Previous UARC criteria were any of the following: leukocyte esterase trace or greater, positive nitrites, or white blood cells (WBC) > 10 cells/high powered field (HPF). On March 15, 2023, the criteria were modified to only WBC > 10 cells/HPF.

**Methods:**

This was a pre-post study evaluating UARCs ordered in the emergency department or inpatient units from March 1 to March 31, 2022 (pre-intervention) and March 16 to April 15, 2023 (post-intervention). The primary outcome was the proportion of reflex UCs prevented. Secondary outcomes included the frequency of repeat UARCs and stand-alone UCs ordered after an initial negative UARC, the incidence of patients with gram-negative rod (GNR) bacteremia within 7 days of a negative UARC, and the rate of ASB treatment in a sample of patients with positive UCs after UARC.

**Results:**

In the pre-intervention, 4761 UARCs were ordered compared to 5420 in the post-intervention; 1806 (37.9%) and 1160 (21.4%) reflexed to UCs, leading to a 43.5% reduction in UCs. The rate of repeat UARCs, stand-alone UCs, and rates of GNR bacteremia with a negative UARC were numerically similar between groups. In the sample of pre-intervention patients (n = 100), 70 were women, median age was 74 years old (IQR 61-82), and median Charlson comorbidity index (CCI) was 5 (IQR 2-6). In total, 83 patients with positive UCs were treated, 43 (51.5%) were asymptomatic. In the post-intervention (n=68), 54 (79.4%) were women, median age was 80 (IQR 64-85), and median CCI was 5 (IQR 4-6). Fifty-five (80.9%) patients were treated, 24 (43.6%) were asymptomatic.

**Conclusion:**

The change in UARC criteria led to a 43.5% relative reduction in UCs. A similar number of patients in the post-intervention and the pre-intervention had GNR bacteremia without a reflex UC. ASB treatment rate was lower in the post-intervention; more studies are needed to determine the true effect of restrictive UARC criteria on ASB treatment.

**Disclosures:**

**All Authors**: No reported disclosures

